# Transthyretin Cardiac Amyloidosis in a Safety-Net Hospital: A Case Series of 10 Afro-Caribbean Patients

**DOI:** 10.1016/j.jaccas.2026.109013

**Published:** 2026-09-23

**Authors:** Keston Rattan, Elizabeth L. Allison, Wayne-Andrew Palmer, Inna Bukharovich

**Affiliations:** a SUNY Downstate Health Sciences University, Brooklyn, New York, USA; b Kings County Hospital Center, Brooklyn, New York, USA

**Keywords:** acute heart failure, cardiac magnetic resonance, echocardiography, genetic disorders, imaging, nuclear medicine

## Abstract

Transthyretin cardiac amyloidosis (ATTR-CA) is profoundly underdiagnosed in Afro-Caribbean populations despite the high prevalence of the Val122Ile (V142Ile) variant (approximately 3.4% carrier frequency among self-identified Black individuals in the United States). This case series describes 10 Afro-Caribbean patients diagnosed with ATTR-CA at a safety-net hospital. Seven patients presented with heart failure symptoms, while 3 presented with alternative manifestations including syncope with high-degree atrioventricular block and pericardial tamponade. The mean age was 75 ± 8 years, with 80% male predominance. All patients demonstrated a positive 99mTc-PYP scan, and cardiac magnetic resonance imaging was performed in 20%. Among patients with completed genetic testing, 50% had the Val122Ile variant, and 50% had the wild-type ATTR. Atrial fibrillation was present in 50%, and 30% required permanent pacemaker implantation for symptomatic bradycardia. This series demonstrates that ATTR-CA can be effectively diagnosed in resource-limited safety-net hospitals, emphasizing these institutions' critical role in reducing health disparities.

## Visual Summary

**Figure:**
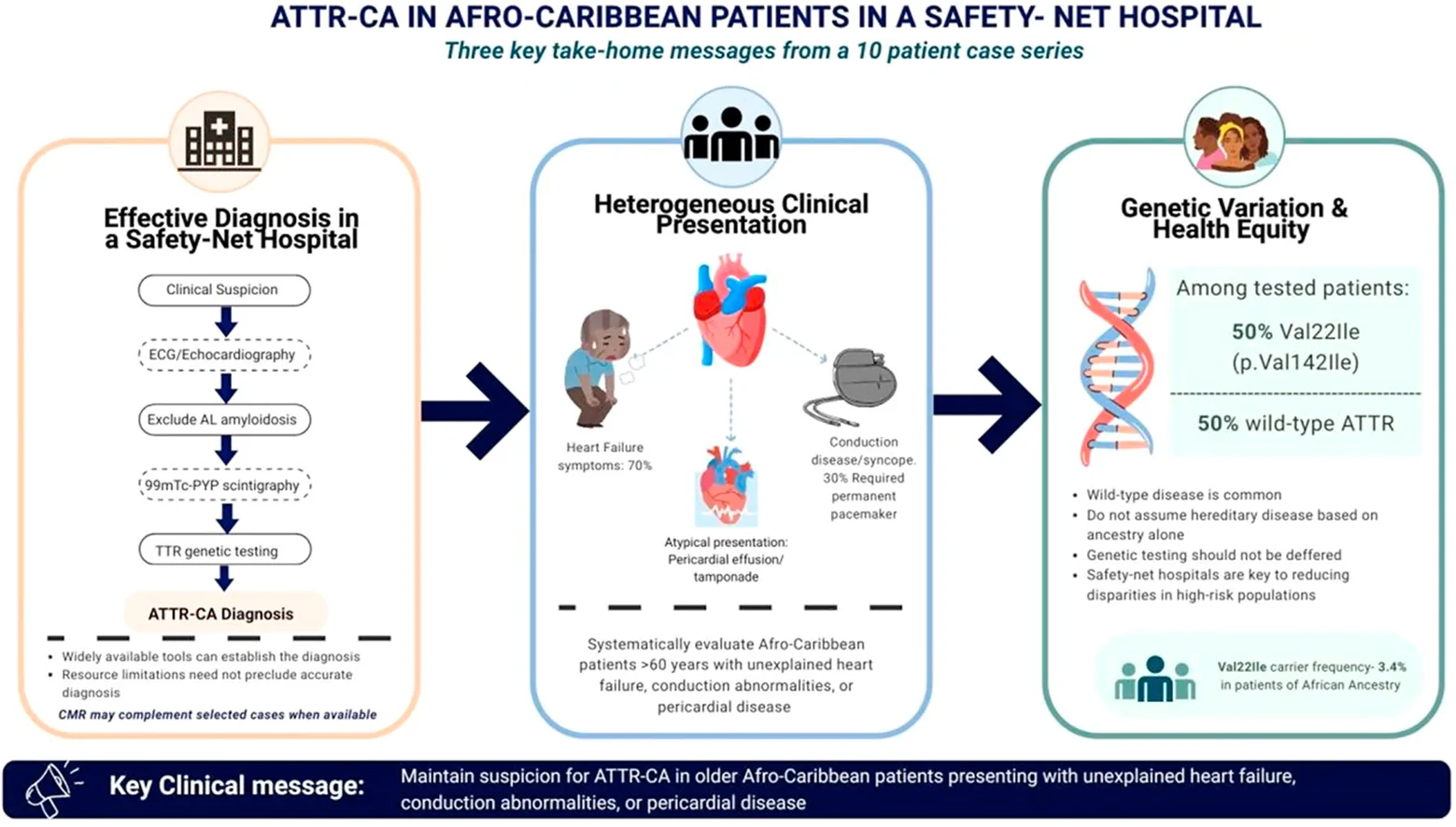

**Visual Summary:**
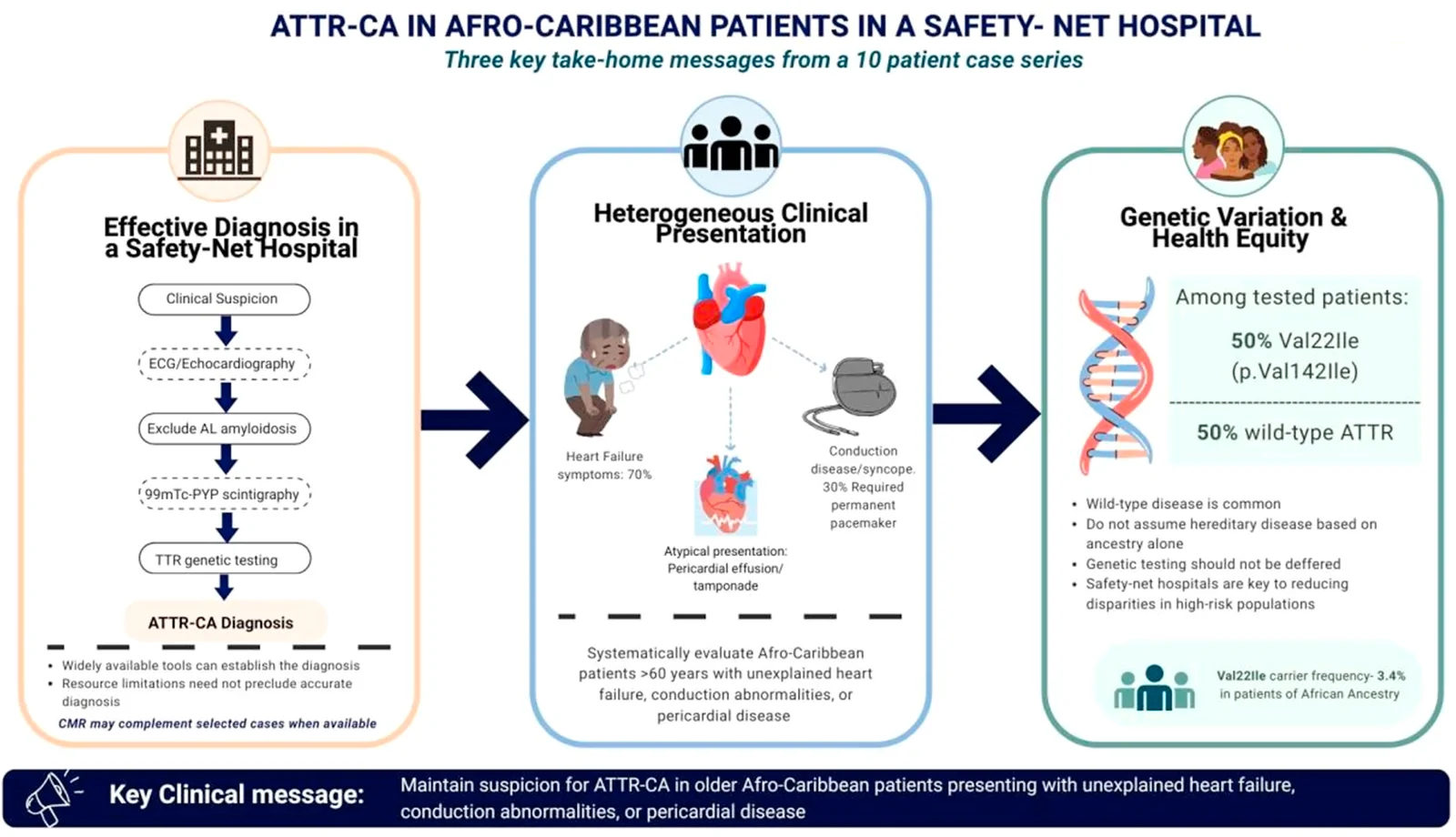
Recognizing ATTR-CA in Afro-Caribbean Patients: Lessons From a Safety-Net Hospital

Transthyretin cardiac amyloidosis (ATTR-CA) results from myocardial deposition of misfolded transthyretin protein, causing progressive restrictive cardiomyopathy and conduction system disease.^1^ The Val122Ile (V142Ile) variant is present in approximately 3.4% of African Americans with similar prevalence in Afro-Caribbean populations; nevertheless, ATTR-CA remains underdiagnosed as highlighted in a large biobank study, where only 10 of 92 Val122Ile carriers with heart failure were evaluated for cardiac amyloidosis.^2^ In addition, the screening for cardiac amyloidosis with nuclear imaging in minority populations (SCAN-MP) study highlights an age-dependent prevalence for ATTR-CA, with a 7.8% prevalence in Black participants with heart failure, increased to 17.2% in age >75; furthermore, wild-type transthyretin amyloidosis (ATTRwt) was more common than variant transthyretin amyloidosis (ATTRv).^3^ Underscoring that in patients with ATTR-CA, V122Ile penetrance is approximately 39% and is age-dependent.^4^

Safety-net hospitals serve disproportionately high numbers of minority and underserved patients with substantial barriers to timely diagnosis of complex conditions, including ATTR-CA. These barriers include limited access to subspecialty care, advanced imaging, and genetic testing, along with social determinants of health. However, advances in multimodality imaging, particularly echocardiography combined with technetium-99m pyrophosphate (PYP) scan, enable noninvasive diagnosis when grade 2 to 3 myocardial uptake occurs in the absence of monoclonal protein.

Described are 10 Afro-Caribbean patients diagnosed with ATTR-CA at a large urban safety-net hospital, highlighting the spectrum of clinical presentations, demonstrating the utility of multimodal imaging in resource-limited settings, and emphasizing the critical role of safety-net hospitals in addressing health disparities in underserved minority populations (Table 1).

**Table 1:** Patient Demographics, Chief Complaint, and Initial Diagnosis on Presentation

Patient	Age (y)	Sex	Ethnicity/Origin	Chief Complaint/Presentation	NYHA Class at Presentation
1	82	M	Jamaica	Shortness of breath heart failure exacerbation	III
2	86	M	Haiti	Cough - Pericardial Tamponade	III
3	80	M	St. Vincent	Generalized Edema - Anasarca	III
4	81	M	Barbados	Syncope – High-degree AV Block	II
5	73	F	Trinidad and Tobago	Dyspnea on exertion heart failure exacerbation	III
6	77	M	St. Vincent	SOB - Pericardial effusion	III
7	66	M	St. Vincent	Shortness of breath - HFrEF	III
8	72	M	St. Vincent	Shortness of breath - HFmrEF	II
9	58	M	Trinidadian and Tobago	Pedal edema	II
10	75	F	Jamaica	Shortness of breath - HFrEF	III
Summary	75 ± 8.4	80% M	100% Afro-Caribbean	70% HF symptoms. 30% conduction/other	70% Class III

HFmrEF = heart failure with mildly reduced ejection fraction; HFrEF = heart failure with reduced ejection fraction.

## Case Presentations

Case 1

#### History and physical

An 82-year-old Jamaican man presented with progressive dyspnea on exertion (NYHA class III) and lower-extremity edema. Medical history included type 2 diabetes mellitus, hypertension, hyperlipidemia, and bilateral carpal tunnel syndrome. Physical examination revealed elevated jugular venous pressure, bibasilar crackles, and 3+ pitting edema. Electrocardiogram showed atrial flutter (AFL), and proBNP was 6,240 pg/mL.

#### Expert commentary

This presentation exemplifies the classic heart failure phenotype of ATTR-CA. The constellation of heart failure symptoms and atrial arrhythmia preceded by a musculoskeletal manifestation, bilateral carpal tunnel syndrome, in an elderly patient represents critical “red flags” that should raise suspicion for infiltrative cardiomyopathy.^1^ In safety-net hospital populations, where hypertensive heart disease is prevalent, distinguishing ATTR-CA requires systematic evaluation. Atrial fibrillation (AF)/AFL occurs in approximately 30% to 70% of ATTR-CA patients due to atrial infiltration and elevated filling pressures, with increased prevalence in ATTRwt when compared to ATTRv.^1^

#### Workup

**Figure 1:**
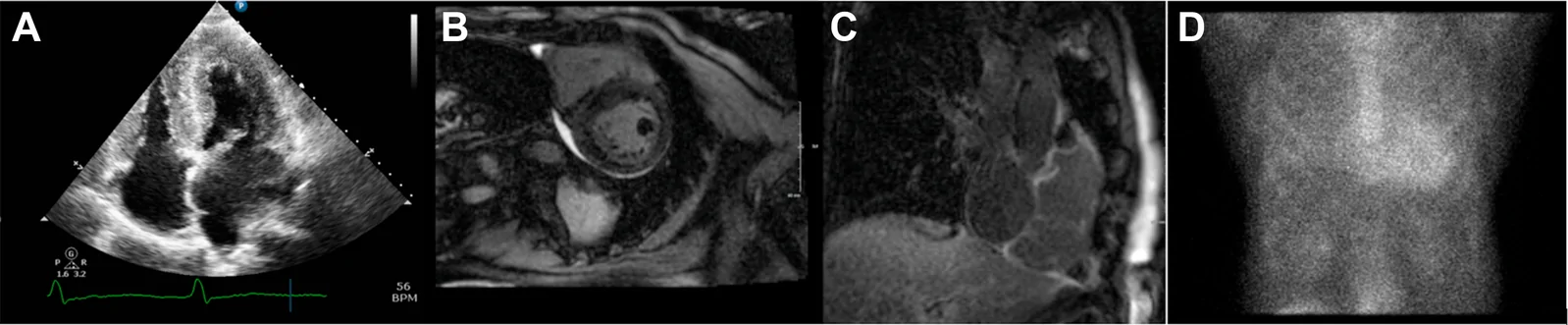
Multimodal Imaging: Echocardiogram, Cardiac Magnetic Resonance Imaging (cMRI) and 99mTc-Pyrophosphate (PYP) Scan (A) Apical 4-chamber view on echocardiogram showing biatrial enlargement, severe concentric left ventricular (LV) hypertrophy and increased LV wall thickness with speckled pattern. (B) Short-axis view on cMRI showing diffuse myocardial wall thickening with increased signal intensity. (C) Sagittal view on cMRI showing extensive late gadolinium enhancement. (D) PYP scan showing significantly increased uptake throughout the left ventricular myocardium, greater than bone marrow activity.

Transthoracic echocardiography (TTE) revealed reduced left ventricular ejection fraction (LVEF) from 35% to 40% with grade III diastolic dysfunction. Serum free light-chain assay and immunofixation electrophoresis were normal, excluding immunoglobulin light-chain amyloidosis (AL) amyloidosis. PYP scan demonstrated positive myocardial uptake (Figure 1).

Given the reduced LVEF and need for comprehensive tissue characterization, cardiac magnetic resonance (CMR) was pursued demonstrating diffuse late gadolinium enhancement consistent with cardiac amyloidosis (Figure 1). Nuclear stress testing showed no stress-induced ischemia; however, there was right ventricle (RV) dilation with increased RV uptake and a mild fixed apical wall defect.

#### Expert commentary

This case illustrates the value of multimodal imaging in diagnosing ATTR-CA. While PYP scan confirms ATTR subtype with a 100% positive predictive value when monoclonal protein is absent, CMR provides complementary information including tissue characterization and assessment of disease burden.^1^^,^^3^ The combination of reduced LVEF, severe diastolic dysfunction, and RV involvement indicates advanced disease. Importantly, CMR, while not essential for diagnosis, was accessible at this safety-net hospital, demonstrating that a comprehensive evaluation is achievable in resource-limited settings.

#### Genetic testing

TTR gene sequencing via mass spectrometry identified the heterozygous Val122Ile variant, confirming ATTRv. The patient was initiated on disease-modifying therapy with tafamidis. Anticoagulation was started for AFL.

Case 2

#### History and physical

**Figure 2:**
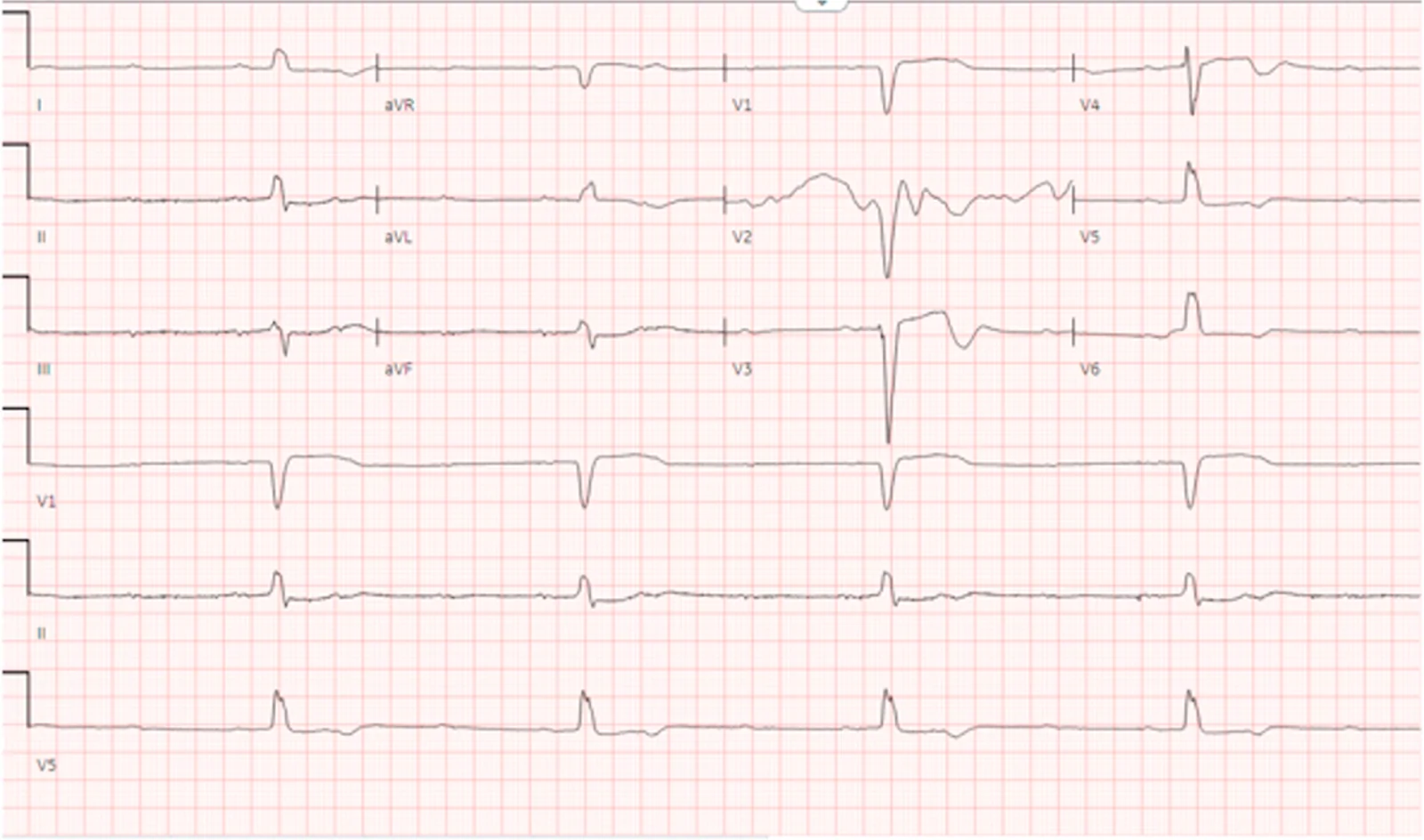
Electrocardiograph Showing Marked Sinus Bradycardia with a Ventricular Rate of 27 Beats/Mini and High-Degree Atrioventricular Block

An 81-year-old Barbadian man presented with syncope. Evaluation revealed high-degree atrioventricular (AV) block requiring urgent permanent pacemaker implantation (Figure 2). Medical history included AF, type 2 diabetes mellitus, hypertension, hyperlipidemia, coronary artery disease, and heart failure with reduced LVEF. He had minimal heart failure symptoms at presentation.

#### Expert commentary

This case illustrates that ATTR-CA may present primarily with conduction system disease rather than overt heart failure symptoms. Amyloid infiltration of the conduction system causes progressive AV block, bundle branch block, and sinus node dysfunction, often requiring pacemaker implantation.^1^ In safety-net populations, the combination of heart failure and unexplained conduction abnormalities in patients older than 60 years, particularly those of African or Caribbean descent, should prompt consideration of ATTR-CA. The presence of AF alongside high-degree AV block further supports infiltrative disease affecting both atrial and conduction system tissue.

#### Workup

TTE revealed LVEF 40% to 45%. Diastolic function was unable to be adequately assessed. Given the clinical suspicion for infiltrative cardiomyopathy in a patient with unexplained conduction disease and AF, further workup was pursued.

Serum free light chains were normal. PYP scan demonstrated positive myocardial uptake, confirming ATTR-CA. Nuclear stress testing was positive for ischemia with a moderate-sized, mildly severe reversible perfusion defect in the basal inferior wall, LVEF 40%, transient ischemic dilation (ratio 1.3), and severe hypokinesis of the basal inferior wall with increased RV uptake.

#### Expert commentary

The coexistence of ATTR-CA and coronary artery disease in this patient highlights the complexity of managing these patients. Transient ischemic dilation and reversible perfusion defects warrant coronary evaluation, and it is worth noting that the presence of coronary artery disease and myocardial scarring from prior infarction may lead to a false-positive PYP uptake. Importantly, ATTR-CA does not preclude concomitant ischemic heart disease, as both conditions require management.^1^ The nuclear stress test findings demonstrate the utility of this widely available modality in safety-net hospitals for comprehensive cardiac assessment.

#### Genetic testing

TTR gene sequencing revealed wild-type sequence, confirming ATTRwt. A permanent pacemaker was implanted. The patient was initiated on tafamidis and anticoagulation for AF.

Case 3

#### History and physical

An 86-year-old Haitian man presented with cough and was found to have pericardial tamponade requiring transfer for urgent pericardiocentesis. This unusual presentation led to a comprehensive cardiac evaluation. Medical history included hypertension and stage 3 chronic kidney disease.

#### Expert commentary

Pericardial effusion is an uncommon but recognized manifestation of cardiac amyloidosis; however, frank cardiac tamponade remains rare. The mechanism involves amyloid infiltration of the pericardium and impaired lymphatic drainage.^5^ This presentation underscores the importance of considering ATTR-CA in the differential diagnosis of unexplained pericardial disease, particularly in elderly patients. Safety-net hospitals frequently manage acute pericardial disease, representing an opportunity for ATTR-CA screening.

#### Workup

Following pericardiocentesis and stabilization, TTE revealed LVEF 45% with grade III diastolic dysfunction, a discordance suggesting infiltrative cardiomyopathy. During pericardiocentesis, an endomyocardial biopsy was performed and confirmed the presence of extracellular amyloid deposits. Serum free light-chain assay and immunofixation electrophoresis were normal. PYP scan demonstrated grade 3 myocardial uptake.

#### Expert commentary

This case demonstrates the value of maintaining a high index of suspicion for ATTR-CA even in atypical presentations. The combination of pericardial tamponade, mildly reduced LVEF, and severe diastolic dysfunction created a clinical picture that could easily be attributed to other etiologies. The systematic approach, excluding AL amyloidosis and confirming ATTR with PYP scan, allowed for timely diagnosis.

#### Genetic testing

TTR gene sequencing revealed wild-type sequence, confirming ATTRwt. The patient was initiated on tafamidis with close monitoring given his advanced age and unusual presentation.

### Cases 4 to 10: clinical spectrum summary

The remaining 7 patients highlight the spectrum of ATTR-CA presentations in Afro-Caribbean populations. Geographic origins included St Vincent (n = 4), Trinidad (n = 2), and Jamaica (n = 1) (Table 1).

#### Presentations

Six patients presented with heart failure symptoms: generalized edema/anasarca (patient 3, 80M, St Vincent), dyspnea on exertion with acute decompensated heart failure (patient 5, 73F, Trinidad), shortness of breath with pericardial effusion (patient 6, 77M, St Vincent), shortness of breath with heart failure with reduced ejection fraction (patient 7, 66M, St Vincent; Patient 10, 75F, Jamaica), and shortness of breath with heart failure with mildly reduced ejection fraction (patient 8, 72M, St Vincent). One patient presented with lower-extremity edema (patient 9, 58M, Trinidad).

### Echocardiographic findings

LVEF ranged from 20% to 50%-55%. Diastolic dysfunction was documented in 4 patients: grade III (severe) in 2, grade II (moderate) in 1, and grade 1 (mild) in 1. Three patients had diastolic function not formally reported (Table 2).

**Table 2:** Multimodal Imaging Performed

Patient	LVEF (%)	Diastolic Dysfunction Grade	99mTc-PYP	Cardiac MRI	Nuclear Stress Test Findings
1	35%-40%	Grade III (severe)	Positive	Yes	No stress-induced ischemia; LVEF 28%; diffuse hypokinesis; RV dilated; mild fixed apical defect
2	45%	Grade III (severe)	Positive	Yes	No nuclear stress test on record
3	44%	Grade II (moderate)	Positive	No	Fixed perfusion defect (outside hospital)
4	40%-45%	Not reported	Positive	No	Positive for ischemia; moderate reversible inferior wall defect; EF 40%; TID 1.3
5	50%-55%	Not reported (HFpEF)	Positive	No	No nuclear stress test on record
6	20%	Unable to assess	Positive	No	EF 34%; TID noted; small fixed apical defect; no stress induced ischemia
7	45%	Not reported	Positive	No	No nuclear stress test on record
8	45%-50%	Grade III (severe)	Positive	No	Normal perfusion; no defects or infarction; low-risk study
9	43%	Not reported	Positive	No	Exercise echo positive for ischemia (wall motion did not improve)
10	25%-30%	Grade I (mild)	Positive	No	Abnormal; no ischemia; small apical scar; global hypokinesis; EF 36%
Summary	39.3% ± 10.2%	Grade III: 40%	100% Positive	20%	Performed in 60%

EF = ejection fraction; HFpEF = heart failure with preserved ejection fraction; LVEF = left ventricle ejection fraction; MRI = magnetic resonance imaging; RV = right ventricle; TID = transient ischemic dilation.

#### Conduction abnormalities

Complete heart block requiring permanent pacemaker placement occurred in 2 patients (patients 3 and 10). First-degree AV block was present in 2 patients (patients 5 and 6). AF was present in 3 patients (patients 3, 7, and 8), with patient 7 demonstrating AF with slow ventricular response (Table 3).

**Table 3:** Arrhythmia and Conduction Disease

Patient	Atrial Fibrillation/Flutter	AV Conduction Disease	Pacemaker Required	Anticoagulation
1	Atrial Flutter	No	No	Yes
2	No	No	No	No
3	Atrial fibrillation	Complete heart block	Yes (PPM)	Yes
4	Atrial fibrillation	High-degree AV Block	Yes (PPM)	Yes
5	No	1st Degree AV Block	No	Yes
6	No	1st Degree AV Block	No	No
7	Atrial fibrillation	No	No	Yes
8	Atrial fibrillation	No	No	Yes
9	No	No	No	No
10	No	Complete heart block	Yes (PPM)	No
Summary	50% (5/10)	50% (5/10)	30% (3/10)	50% (5/10)

#### Imaging

All 7 patients demonstrated positive PYP scans. Nuclear stress testing was performed in 4 patients with variable findings: normal perfusion in 1, fixed defects without ischemia in 2, and positive for ischemia on exercise echocardiography in 1 (Table 2).

#### Genetic testing

TTR gene sequencing was completed in 5 of 7 patients. The Val122Ile (p.Val142Ile; c.424 G>A) variant was identified in 3 patients (patients 7, 8, and 9). ATTRwt was confirmed in 2 patients (patients 6 and 10). Testing remains pending in 2 patients (patients 3 and 5). Notably, patient 9 (58M, Trinidad) had compound genetic findings with both TTR Val122Ile and PCCB c.990dup (p.Glu331) mutations, representing the youngest patient in the cohort (Table 4).

**Table 4:** Genetic Testing

Patient	TTR Genetic Testing	Variant Identified	Testing Method	ATTR Type	Family Screening Indicated
1	Completed	Val122Ile (p.Val142Ile)	Mass spectrometry	Hereditary (ATTRv)	Yes
2	Completed	None (wild-type)	Mass spectrometry/LC-MS/MS	Wild-type (ATTRwt)	No
3	Not performed	—	—	—	TBD
4	Completed	None (wild-type)	Gene sequencing	Wild-type (ATTRwt)	No
5	Not performed	—	—	—	TBD
6	Completed	None (wild-type)	Gene sequencing	Wild-type (ATTRwt)	No
7	Completed	Val122Ile (p.Val142Ile; c.424G>A)	Gene sequencing	Hereditary (ATTRv)	Yes
8	Completed	Val122Ile (p.Val142Ile; c.424G>A)	Gene sequencing	Hereditary (ATTRv)	Yes
9	Completed	Val122Ile (p.Val142Ile; c.424G>A) + PCCB c.990dup	Gene sequencing	Hereditary (ATTRv)	Yes
10	Completed	None (wild-type)	Gene sequencing	Wild-type (ATTRwt)	No
Summary	80% Completed	Val122Ile: 50% (of tested)	—	ATTRv 50%; ATTRwt 50%	50% of tested require screening

#### Expert commentary

The clinical heterogeneity in this cohort, ranging from heart failure with preserved ejection fraction to heart failure with reduced ejection fraction, with and without conduction disease, and variable diastolic dysfunction severity, underscores that ATTR-CA should be considered across the spectrum of heart failure phenotypes in at-risk populations. The 50% prevalence of AF and 30% pacemaker requirement align with published literature on conduction system involvement in ATTR-CA.^1^ The identification of both ATTRwt and ATTRv in this Afro-Caribbean cohort (50% each among those tested) is consistent with the SCAN-MP study which found that wild-type disease is not uncommon in Black patients with ATTR-CA.^1^^,^^3^^,^^4^

## Discussion

This case series demonstrates that ATTR-CA in Afro-Caribbean patients presents through diverse clinical pathways: heart failure symptoms, conduction abnormalities, and atypical manifestations including pericardial tamponade (Table 5). Among patients with completed genetic testing, the distribution was 50% ATTRv and 50% ATTRwt, highlighting that hereditary disease should not be assumed in this population. Furthermore, ATTR-CA can be effectively diagnosed in safety-net hospital settings using widely available imaging modalities.

**Table 5:** Summary of Patient Cohort

Pt	Age/Sex	Origin	Presentation	LVEF	Diastolic Dysfunction	TTR Variant	Atrial Fibrillation/Flutter	Conduction Disease	PPM
1	82/M	Jamaica	Heart failure exacerbation	35%-40%	Grade III	Val122Ile	Atrial flutter	No	No
2	86/M	Haiti	Pericardial tamponade	45%	Grade III	Wild type	No	No	No
3	80/M	St. Vincent	Anasarca	44%	Grade II	Pending	Atrial fibrillation	Complete heart block	Yes
4	81/M	Barbados	Syncope/AV Block	40%-45%	Not reported	Wild type	Atrial fibrillation	High-degree AVB	Yes
5	73/F	Trinidad and Tobago	Heart failure exacerbation	50%-55%	HFpEF	Pending	No	1° AVB	No
6	77/M	St. Vincent	Pericardial Effusion	20%	Unable	Wild type	No	1° AVB	No
7	66/M	St. Vincent	Heart failure exacerbation	45%	Not reported	Val122Ile	Atrial fibrillation	No	No
8	72/M	St. Vincent	Heart failure exacerbation	45%-50%	Grade III	Val122Ile	Atrial fibrillation	No	No
9	58/M	Trinidad and Tobago	Pedal Edema	43%	Not reported	Val122Ile + PCCB	No	No	No
10	75/F	Jamaica	Heart failure exacerbation	25%-30%	Grade I	Wild type	No	Complete heart block	Yes

HFpEF = heart failure with preserved ejection fraction

## The Role of Safety-Net Hospitals in Addressing Health Disparities

Safety-net hospitals serve as the primary source of care for millions of minority and underserved patients in the United States. These institutions are uniquely positioned to address the profound underdiagnosis of ATTR-CA in Afro-Caribbean populations for several reasons. First, they serve the populations at the highest risk; the Val122Ile variant is present in 3.4% of African Americans and similarly prevalent in Afro-Caribbean populations.^1^ Second, these hospitals frequently manage patients with heart failure, conduction abnormalities, and acute presentations like pericardial tamponade, representing opportunities for systematic ATTR-CA screening. Third, the diagnostic tools required such as echocardiography, PYP scan, and serum free light-chain assays are available in most safety-net hospital settings.

The geographic diversity of our cohort reflects the Caribbean diaspora served by urban safety-net hospitals, underscoring the importance of recognizing ATTR-CA risk across Afro-Caribbean populations, not solely African Americans.

## Diagnostic Approach in Resource-Limited Settings

Echocardiography serves as the initial screening tool, with findings of increased wall thickness, diastolic dysfunction, and reduced global longitudinal strain raising suspicion for cardiac amyloidosis. Exclusion of AL amyloidosis is essential and widely available. PYP scan, available in most nuclear medicine departments, confirms ATTR-CA when positive uptake is present in the absence of monoclonal protein, with a 100% positive predictive value.^1^ This noninvasive pathway obviates endomyocardial biopsy.

CMR can be accessed in safety-net settings when clinically indicated; though it is worth noting that it may not be readily available in all safety-net hospitals. The workup and subsequent diagnosis of ATTR-CA in the absence of CMR may be approached using a multimodality approach including high clinical suspicion, electrocardiogram, TTE, AL exclusion, PYP scan, and genetic testing. Nuclear stress testing may provide valuable information regarding concomitant ischemic heart disease.

TTR gene sequencing, while not universally available in safety-net hospitals, can be obtained through commercial laboratories and is essential for distinguishing ATTRv from ATTRwt, informing prognosis, and guiding family screening.^1^^,^^2^ The finding that 50% of our tested patients had wild-type disease emphasizes that genetic testing should not be deferred based on assumptions about hereditary disease in Afro-Caribbean patients.

## Clinical Implications

The high burden of conduction disease in our cohort, 50% with AF/AFL and 30% requiring permanent pacemaker, has important management implications. All patients with AF should receive anticoagulation regardless of the CHA_2_DS_2_-VASc score given the high thromboembolic risk in cardiac amyloidosis.^1^ Pacemaker implantation should prompt ATTR-CA evaluation in at-risk patients, and device selection should consider potential future need for biventricular pacing.

The coexistence of ischemic heart disease in several patients (positive stress testing in 3 of 6 tested) highlights that ATTR-CA does not preclude concomitant coronary disease, and comprehensive cardiovascular evaluation remains essential.

The mean time from diagnosis to disease-modifying therapy with tafamidis in this patient cohort was approximately 7 months due to institutional workflow limitations, highlighting the need for streamlined management.

## Funding Support and Author Disclosures

The authors have reported that they have no relationships relevant to the contents of this paper to disclose.

**Take-Home Messages**

• ATTR-CA can be effectively diagnosed in safety-net hospitals using widely available imaging modalities (echocardiography and 99mTc-PYP scintigraphy), demonstrating that resource limitations need not preclude accurate diagnosis in high-risk Afro-Caribbean populations.
• The clinical presentation of ATTR-CA extends beyond classic heart failure to include conduction abnormalities (30% required pacemaker) and atypical manifestations such as pericardial tamponade; any Afro-Caribbean patient over the age of 60 with unexplained heart failure, conduction disease, or pericardial disease should be systematically evaluated.
• Among Afro-Caribbean patients with ATTR-CA, wild-type disease is common (50% in this series), emphasizing that genetic testing should not be deferred based on assumptions about hereditary disease, and safety-net hospitals are uniquely positioned to address health disparities given their role serving minority populations at highest risk.
